# Characterization of raw material in textile bone instruments from the capital of the Roman Province of Lusitania (Mérida, Spain)

**DOI:** 10.1038/s41598-026-55935-6

**Published:** 2026-06-01

**Authors:** Roshan Paladugu, Mariya Antonosyan, Macarena Bustamante-Álvarez, Patrick Roberts, Cleia Detry

**Affiliations:** 1 Department of Evolutionary Genetics & aDNA Sequencing Core Unit, Max Planck Institute for Evolutionary Anthropology, Deutscher Platz 6, 04103 Leipzig, Germany; 2 Department of History, School of Arts and Humanities, Uniarq-Center of Archaeology of the University of Lisbon, University of Lisbon, Alameda da Universidade, Lisboa, 1600-214 Portugal; 3 Department of Coevolution of Land Use and Urbanisation, Max Planck Institute of Geoanthropology, 07745 Jena, Germany; 4 Departamento de Prehistoria y Arqueología, Facultad de Filosofía y Letras, University of Granada, Campus Universitario de Cartuja, Granada, 18071 Spain

**Keywords:** Craftmanship, Collagen, Peptide mass fingerprinting, Iberia, Ecology, Ecology, Evolution, Zoology

## Abstract

Bone has long served as a versatile raw material in human societies due to its durability, workability, and availability. However, identifying the animal species used in worked bone artifacts remains challenging, as manufacturing processes and use-wear often remove diagnostic morphological features. This study applies Zooarchaeology by Mass Spectrometry (ZooMS) to investigate raw material selection in textile-related bone tools from Augusta Emerita (Mérida, Spain), the capital of Roman Lusitania, dating to the 1st − 3rd centuries CE. The archaeological context of the assemblage is particularly significant, originating from contexts consisting of a bone processing workshop for the manufacture and sale of bone objects that is one of the first installations of this type identified in the Iberian Peninsula. A total of 36 artifacts associated with textile production were analyzed where five main taxonomic groups were identified: *Bos taurus*, Bovidae/Cervidae, *Cervus elaphus*, *Elephas maximus*, and an unidentified avian species. These results indicate a functional selection of common domestic and wild taxa, while the presence of exotic materials points to long-distance trade networks and the potential symbolic or social significance of certain artifacts, particularly in funerary contexts.

**Supplementary Information:**

The online version contains supplementary material available at 10.1038/s41598-026-55935-6.

## Introduction

Due to its durable nature, combined with excellent malleability, bone has served, and continues to serve, as a key raw material for a wide range of purposes, from personal ornaments to craft-related activities. This versatility extends across various cultures and time periods throughout human history. Bone’s role as a material of choice is well documented from the Upper Palaeolithic onwards, with examples ranging from hunting weapons and sewing needles to symbolic objects and musical instruments^1,2^. Its combination of strength, lightweight properties, and ease of modification has ensured its widespread use across both prehistoric and historic contexts. During the Roman period, bone constituted an important raw material within urban craft production and was widely used for the manufacture of everyday objects such as pins, needles, spindle whorls, handles, gaming pieces, and decorative items, often produced in specialized workshops^3,4^. The study of bone artifacts in Roman contexts therefore provides valuable information on craft organization, urban economies, and systems of raw material procurement and distribution. This is particularly significant in Lusitania, and especially in *Augusta Emerita* as the provincial capital, where local production activities coexisted with long-distance trade networks, offering an important framework for investigating patterns of resource selection and craft specialization.

Archaeological research has extensively examined bone artifacts, primarily focusing on their operational sequences (*chaîne opératoire*) and production locales^5^, as well as the typological classification of the finished products (from the pioneering study by J.C.Béal^6^, to the more recent work by^7^. These studies have provided significant insights into manufacturing techniques, cultural preferences, and the social and economic roles of bone artifacts. The principal challenges hindering raw material source identification stem from the inherent limitations of worked bone. The first limitation concerns the preparation processes required for bone manufacturing. These involve a series of preliminary treatments, including the removal of residual soft tissues by immersion in corrosive substances. Classical textual sources, such as Plutarch (*An vitio sitas ad infelicitatem sufficia*,* IV*), mention the use of ashes or vinegar for this purpose, while later scholars suggest other materials like spoiled milk^8^ or quicklime^9^. Archaeological findings from the only bone-working workshop in Hispania with preserved architectural features^5^ support these textual accounts, as evidenced by the discovery of vats bearing residues of adhered lime. Such chemical treatments, however, alter the biochemical properties of the bone, potentially thwarting species identification through both morphological and molecular methods^10^. The second major limitation lies in the technical processes involved in crafting bone artifacts. Operations such as rough shaping, sawing, polishing, and dyeing, common in bone tool production, gradually remove diagnostic anatomical features that would otherwise aid in determining the species origin^11^. A further complicating factor arises from the functional nature of many bone artifacts. Items such as textile tools (e.g., spindle whorls, weaving implements), which are the focus of this study, often exhibit use-wear patterns that mask or distort traces of the original raw material. Such wear includes smoothing, striations, or pigment stains, all of which can obscure species-specific traits^12,13^.

Nevertheless, it remains common practice to attribute worked bone to certain species, with suinae, cervids, equines, caprines (sheep and goats), and bovines being among the most frequently identified taxa. This attribution often relies on the selection of specific skeletal elements such as metacarpals, metatarsals, humeri, femora, tibiae, radii, or ulnae which are particularly suited for crafting (Béal, 1983: 20–21)^6^. Such attributions are relatively straightforward when the raw material is found unworked or in the early stages of the *chaîne opératoire*. In contrast, once the artifacts are fully processed and separated from associated production waste, accurately determining the species of origin becomes significantly more challenging. The identification of the species used in bone tool manufacture has only been possible with molecular methods such as aDNA and paleoproteomics^12,14–19^. However, ancient DNA analyses are often expensive especially for a large number of samples. Proteins have proven to be effective alternative to DNA for taxonomic identification. Peptide mass fingerprinting and tandem mass spectrometry (LC-MS/MS)-based methods such as ZooMS and Species by Proteome INvestigation (SPIN), can reliably identify the taxa using the differences in collagen type I (COL1). ZooMS is a reliable, low-cost technique that uses tryptic digests of bone or other collagen-containing tissues and a matrix-assisted laser desorption/ionization time-of-flight (MALDI-ToF) mass spectrometer to generate peptide mass fingerprints.

In this study, we apply a multi-method approach to precisely identify the raw materials of tools associated with the textile industry. Specifically, we combine traditional morphological characterization with ZooMS identification of animal species of the bones used for manufacture. This methodology enables us, on the one hand, to scientifically confirm morphological estimates and, on the other hand, to make it possible to identify species not previously considered in this type of production.

## Background of the repertoire

Within the framework of the project *Texlus. La economía del artesanado textil en la Lusitania romana* project, an exhaustive collection of various instruments used for textile craft activity has been assessed. The fundamental objective of *Texlus* is to examine textile craftsmanship in the province of Lusitania, located on the Spanish Atlantic coast between the 1st and 3rd centuries CE. This survey encompasses a broad spectrum of instruments (not only in bone, but also in metal and glass), ranging from tools employed in the procurement of raw materials to needles intended for the repair of finished textile products. However, the predominant material documented, accounting for over 90% of the sample, which exceeds 2,000 artifacts, is bone. This prevalence raised questions regarding the specific animal species selected for manufacturing these objects. Given that these items originated from contexts of use, dumps, or funerary areas, and thus represent finished products without association to other diagnostic bone fragments that could provide more reliable data, hampering species determination.

**Fig. 1:**
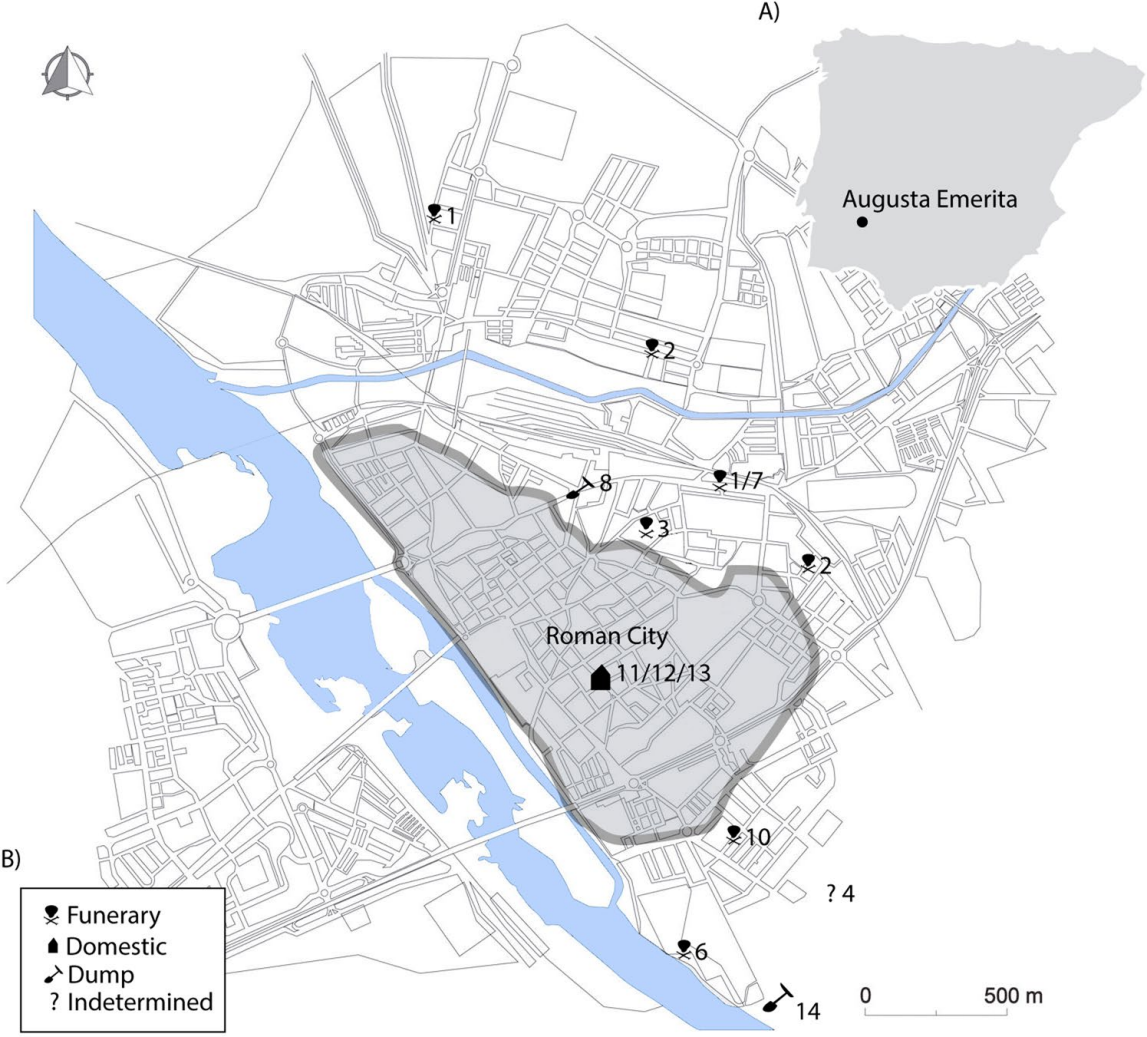
Location of Mérida on the Iberian Peninsula and layout of the ancient city with indication of the contexts analyzed. 1.- Cabo Verde St., Pontezuelas St., and Octavio Augusto St.: El Disco; 2.- Former Campsa site: Campsa; 3.- Pontezuelas St. / Travesía Rambla Sta. Eulalia / J.R. Mélida; 4.- Tomás Romero de Castilla St. and Antonio Hernández Mancha St.; 5.- Vía de la Plata Avenue / Albañiles St.; 6.- Lusitania Avenue / Barcelona St. and José Echegaray St.; 7.- Cabo Verde St. and Hernán Cortés St. 8.- Almendralejo, 41. Str; 9.- Corchera Road; 10.- House of the amphitheater: Mausoleum; 11.- Parejo St.; 12.- “Travesía” Parejo, at the corner of “Travesía” Hernán Cortés; 13.- Hernán Cortés Sr. and 14.- Zone III. Antonio García Bellido St.

**Fig. 2:**
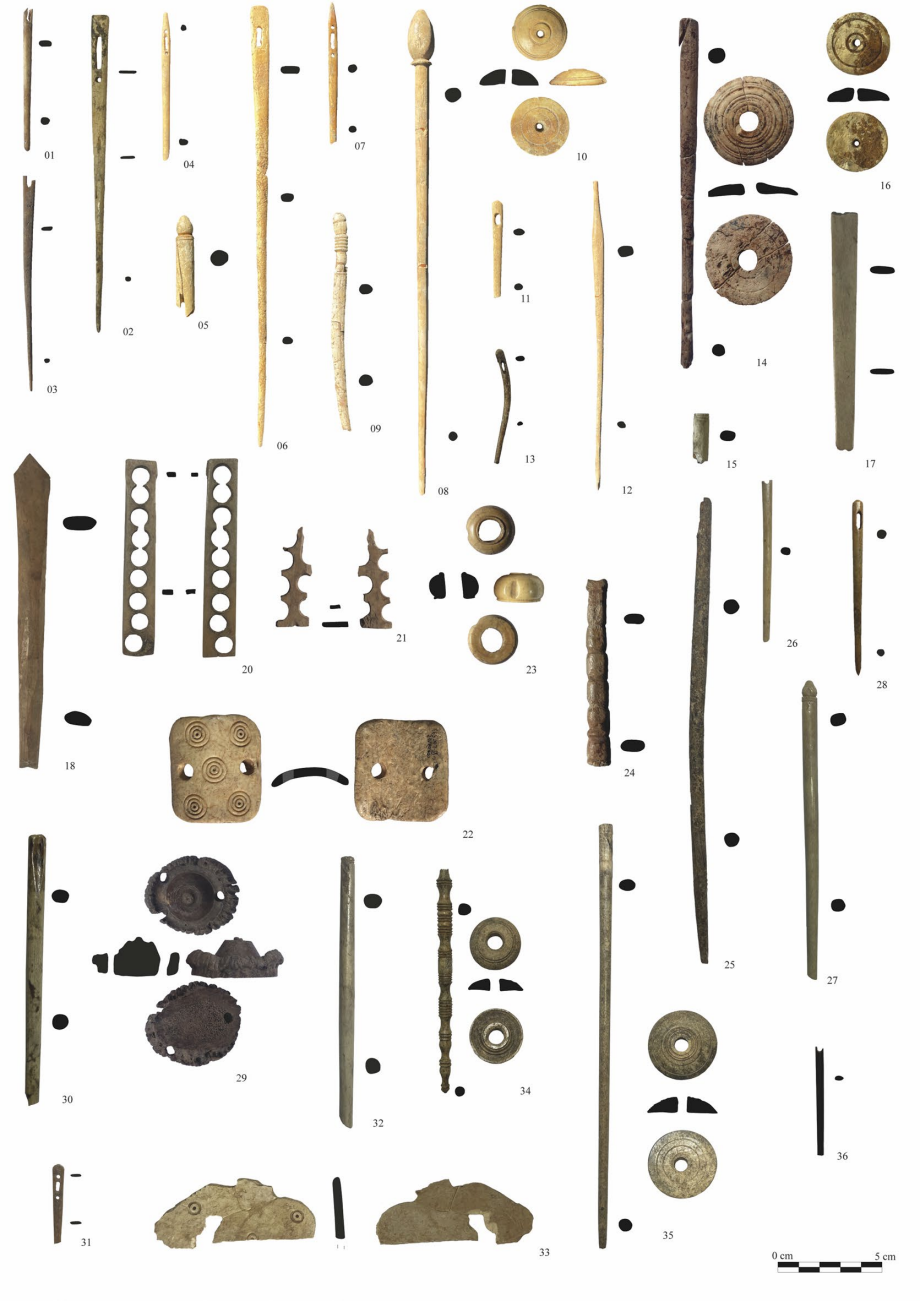
The selected 36 textile crafting bone tools from the larger assemblage of *Augusta Emerita*. Though present in this figure, access to sample MER19 could not be secured for ZooMS analysis. The original sample numbering was retained for consistency within the project’s broader scope.

Here we focus on an assemblage from *Augusta Emerita* (Fig. 1), the capital of the Roman province of Lusitania (current Mérida, Spain). Administratively, this place was the capital of one of the provinces that were formed on the Iberian Peninsula, Lusitania. The site has provided preliminary insights into the most suitable raw materials used for textile crafting during the Roman period. An earlier zooarchaeological study focusing on a workshop (officina) at Casa de Mitreo (Mérida) and for the production and sale of bone objects, was able to confirm the use of the species *Bos taurus* (cattle), *Ovis aries* (sheep), *Capra hircus* (goats), *Sus* sp. (pigs or wild boars), *Cervus elaphus* (red deer), *Equus* sp. (horses or donkeys), *Oryctolagus cuniculus* (rabbits), *Capreolus capreolus* (roe deer), and *Camelus* sp. (camels)^5^, Fig. 8). In relation to the nature and function of the contexts in which they were found, we can characterize them as heterogeneous (For more specific data, see Figs. 1 and 2, and Supplementary Table 1).

## Results

Of the 35 bone artifacts analyzed, collagen was successfully extracted from 32 specimens (91.4%), demonstrating a high level of protein preservation across the assemblage. Only 3 specimens (MER1, MER13, MER15) failed to yield sufficient collagen for analysis, likely due to poor molecular preservation. To ensure data integrity, extraction blanks were incorporated at all stages of the protocol to monitor for potential contamination. These blanks consistently tested negative for type I collagen, confirming that no exogenous protein contamination was introduced during laboratory procedures.

Four mammalian and one avian taxonomic groups were identified through ZooMS analysis. The outcomes of the taxonomic identifications are summarized in Table 1, with a detailed list of the ZooMS peptide markers provided in Supplementary Table 2. The identified taxa include both autochtothonous and exotic species, indicating a broad selection of faunal resources exploited for bone material production.

In the majority of cases, identifications were made at the genus level (*n* = 27; 84.3% of the identified specimens). However, in a subset of samples, limited collagen preservation constrained identifications to the family level (*n* = 4; 12.5%). For example, four specimens (MER2, MER8, MER36, and MER27) could only be assigned to the category “Bovidae/Cervidae” due to poor collagen preservation (Table 1). Bovines dominates the assemblage, comprising 65.62% of the identified assemblage. This is followed by *Cervus elaphus* at 15.62%, Bovidae/Cervidae at 12.5%, and finally *Elephas maximus* and unidentified avian, each represented by a single specimen (3.12%).

The taxonomic resolution achievable through ZooMS is dependent on the evolutionary divergence between taxonomic groups^20^. While differentiation at the family level and, in many cases, at the genus level can be performed with a high degree of confidence, distinguishing between closely related species remains challenging due to the limited variability in collagen peptide sequences^21^. For instance, *Bos* sp. and *Bison* sp. can be reliably distinguished from other artiodactyls through a specific combination of *m/z* values: 1192.6 and 1208.6 (COL1A2 978–990), 1427.7 (COL1A2 484–498), 2853.4 (COL1A1 586–618), and 3033.4 (COL1A2 757–789). However, these peptide markers do not differentiate between *Bos* sp. and *Bison* sp., as their tryptic collagen fingerprints are identical. In such cases, taxonomic identification must rely on biogeographical and archaeological context, considering both current and historical faunal distributions. In this study, 18 artifacts were assigned to *Bos taurus* (Table 1) since *Bos primigenius* has not been reported in Iberia after the Iron Age, nor in Mérida^22^. We also excluded *Bison* sp. as a potential source, given that this genus has not been recorded in faunal assemblages from the Roman period in Europe. In contrast, the use of ZooMS enabled to identify 5 artifacts as *Cervus elaphus*, which can be reliably distinguished from other members of Cervidae through the detection of a unique diagnostic peptide, COL1A1 910 at *m/z* value 2216^23,24^.

One specimen, a spinning wheel (MER25), was assigned to the Elephantidae family based on the presence of a specific combination of *m/z* values: 1453 (COL1A2 484–498), 2115 (COL1A2 793–816), 2853 and 2869 (COL1A1 586–618), along with 2999.4, and 3015.4 (COL1A2 757–789). Additionally, the detection of marker peaks at *m/z* 1568.8 (COL1A2 292–309) and 1590.8 (COL1A2 889–906) provided further resolution, allowing the specimen to be confidently identified as *Elephas maximus*. Another unique find is a needle (MER31) made of bird bone, which could not be previously recognized as such solely from its morphological characteristics. The ZooMS reference markers for avians are not fully developed, which restricts precise taxonomic attribution.

## Discussion

Osseous artifacts, including tools and personal ornaments, provide a valuable window into technological practices, resource exploitation, cultural expressions, and socio-economic contexts^25–29^. Identifying the animal species used in the production of these artifacts can provide insights into the socio-economic and symbolic roles animals played in ancient societies. Additionally, it can reveal the local fauna available at the time, contributing to our understanding of regional biodiversity and human-environment interactions. All analyzed specimens in this study correspond to tools associated with textile production, originating from various contexts, predominantly funerary contexts or refuse deposits. The sample was selected randomly, with the dual aim of including artifacts representative of the different stages of the *chaîne opératoire* to explore potential patterns in raw material selection, and of prioritizing specimens that were fragmentary or contained removable flakes suitable for sampling. This approach facilitated the creation of a sample set with minimal selection bias.

The vast majority of the pieces (MER01-MER12, MER14-MER18, MER21, MER22, and MER25) come from funerary contexts and, specifically, some of them were found as part of the grave goods that accompanied the deceased to the afterlife. Similarly, it is important to note that, in some cases, they show signs of thermal alteration, indicating that they were even included in the funeral pyres (Ex: MER 15). The relatively high success rate of ZooMS analysis in this study suggests that the overall thermal impact on the tools was generally below 300 °C, considerably lower than the temperatures typically required for the cremation of human remains^30^. The pieces were therefore most likely exposed to the lower temperatures, either because they were positioned away from the center of the pyre or due to the difficulty of maintaining consistently high temperatures for prolonged periods during ancient cremation practices^30,31^. This may also explain the lower taxonomic resolution achieved for most specimens, often limited to the Family level, as a result of the degradation or loss of more diagnostic species-specific peptides.

This evidence provides us with a strong proof of the attachment and symbolism of these pieces with the deceased. Secondly, the artifacts from urban dumps (MER20, MER26-MER36) allow us to trace the functional aspect despite lacking votive connotations of these pieces. Without a doubt, these pieces were necessary within the textile production chain and, specifically, in activities that involved strong tension forces which, together with their fragility, ended up breaking them. All the pieces in the current study are finished artifacts, except for sample MER29, which is an unfinished spindle whorl, still in the process of being shaped, is of added interest as it helps us to gain a deeper understanding of the process involved in producing these pieces. Finally, we have examples that appear within the domestic spaces (MER23 and MER24). In this case, the idea of craftsmanship confined to the home is reinforced, with women playing a fundamental role.

The successful identification of the 94% of the sampled artifacts through ZooMS, highlights its efficacy and the good preservation condition of the artifacts. Five main taxonomic groups of raw materials were revealed: *Bos taurus*, Bovidae/Cervidae (unresolved bovids or cervids), *Cervus elaphus* (red deer), *Elephas maximus* (Asian elephant), and an unidentified avian species. All of these taxa, with the exception the Asian elephant, are species that are commonly found across the Iberian Peninsula and have been documented in previous zooarchaeological studies^5,32^. The identification of a distaff (MER25) crafted from osteological remains of an Asian elephant is particularly interesting. Widespread use of exotic animals for the purposes of leisure and entertainment in Roman societies is well-documented^33^. The exotic fauna was primarily secured through military hunters and specialized merchants who either sold or leased the animals^34^. Despite the geographical distance of the Roman empire from Asia, the Romans first encountered Asian elephants in their conflict against Pyrrhus in 280 BCE^35^. The Romans, however, showed little interest in using elephants for warfare and were more interested in their ivory and potential as show animals^36^. However, procuring Asian elephants and their ivory proved to be a challenge through overland routes and was only achieved after the establishment of sea trade routes between the Indian subcontinent and Red Sea ports^35,37^. The finding of Asian elephant osteological material clearly supports the possibility that the site was part of a long-distance trade network. Given the logistical burden of transporting a live individual, either Asian elephant bones or ivory was likely imported as a raw material for crafting. However, the potential use of Asian elephants in public spectacles held in one of the city’s three entertainment venues: the circus, theatre, or amphitheatre cannot be totally ruled out. The use of animals from *ludi* (games and spectacles) as a source of raw material is also another likely scenario that has already been considered in the specific case of *Augusta Emerita*^5^. Notably, a humerus fragment from *Camelus* sp., another non-native species, was recovered during excavations at the bone-working workshop in the Domus of the Mithraeum^5^, Fig. 8). Camelids, like elephants, appear to be associated with such performance spaces, a connection further supported by their discovery in other Roman entertainment contexts, such as in the amphitheatre of Cartagena^38^.

It is also important to bear in mind that some of the pieces, such as spindles or distaffs, have a symbolic character beyond their function, as reflected in their participation in major social events such as marriage and death. Classical authors such as Pliny (Nat. Hist. VIII, 194) recorded that brides carried a spindle and a distaff in wedding processions^39^. The exotic source material of the tools reflected the social status, role, and kinship dynamics of the women wielding them, in both life and death^40–42^.

Additionally, the identification of avian bone in one specimen highlights on one hand the broad spectrum of faunal resources utilized and on the other hand the current limitations of ZooMS reference databases. The incomplete development of avian collagen libraries restricts precise taxonomic identification, highlighting the need for expanding reference collections to encompass a wider array of species, particularly non-mammalian taxa.

This study, although based on a limited sample size, provides new insights into raw material selection in Roman Lusitania’s osseous industry, focusing specifically on textile-related bone tools, which have rarely been analyzed for their taxonomic composition. The identification of domestic, wild, and exotic species reflects a complex interplay of availability, functionality, and social or symbolic considerations. The presence of elephant bone or ivory highlights *Augusta Emerita’s* integration into long-distance trade networks and the potential social value of certain artifacts. ZooMS proved a valuable complement to traditional morphological analysis, enabling precise taxonomic identification and enhancing interpretations of craft production, resource use, and human-animal interactions. By linking these data to the bone-working installation in the *Augusta Emerita*, the study sheds light on craft specialization, urban economic organization, and material culture in a Roman provincial capital. Overall, this work demonstrates the potential of biomolecular approaches to refine our understanding of Roman bone industries and their broader cultural and economic significance, particularly in understudied artifact categories such as textile tools.

## Methods

### Samples

We selected 36 artifacts (Fig. 2) recovered in fragmentary condition. The assemblage includes (Table 1): 13 needles, 2 spatulas, 3 spindle whorls, 2 distaff, complete spindle sets including their spindle whorls, spinning wheels, 2 separating tablets, and handlooms originating from diverse contexts (Fig. 1) including landfills, necropolises, and craft workshops implying different functional roles.

**Table 1:** Summary of the studied textile artifacts and their raw material taxonomic identification through ZooMS.

Artifact type	Avian	Bos	Bovidae/Cervidae	Cervus	Elephas
Needles	MER31	MER03, MER04, MER07, MER26, MER28	MER02, MER36	MER06, MER11	–
Looms	–	MER22, MER33	–	–	–
Spatulas	–	MER17, MER18	–	–	–
Separators	–	MER20, MER21	–	–	–
Spindles	–	MER12, MER14, MER35	–	–	–
Distaff	–	MER05, MER24, MER32	MER08, MER27	MER09, MER30	MER25
Spinning wheels	–	MER10, MER16, MER23, MER34	–	MER29	–
Total	1	21	4	5	1

### Collagen extraction and digestion

Samples were processed for ZooMS analysis at the Max Planck Institute for Evolutionary Anthropology and the Max Planck Institute of Geoanthropology using the acid insoluble and acid soluble protocols published earlier^20^.

5 mg of sample was extracted from the broken edges of the artifacts using clippers and transferred to a 2 mL microcentrifuge tube. The sampled bone chunk was demineralized in 500 µL of 0.6 M hydrochloric acid (HCl) for 48 h. The samples were centrifuged and the acid supernatant was pipetted out and stored at -20 °C for the acid-soluble method. The samples were rinsed three times with 200 µL of 50 mM AmBic buffer solution (Ammonium bicarbonate NH_4_HCO_3_; pH 8) to achieve neutral pH. The samples were subsequently gelatinized in 100 mL AmBic at 65 °C and allowed to cool down to room temperature. 50 µL of the collagen extract is transferred to a new 0.5 mL tube and digested with 1 µL of trypsin solution (0.4 µL/µg Thermo Scientific) overnight at 37 °C. The trypsin digestion was stopped by adding 1 µL of 5% TFA (trifluoroacetic acid). For the acid-soluble method, 250 µL of the acid supernatant was loaded onto a 30 kDa ultrafilter and centrifuged at 3700 rpm until the acid had been pushed through the filter. The samples were rinsed twice with 50 mM AmBic was added to the filter and collagen was resuspended using a pipette. The collagen-containing AmBic solution was transferred to a clean 0.5 mL tube for trypsin digestion in the same way as the acid insoluble method.

### MALDI-ToF (matrix-assisted laser desorption/ionization−tandem time-of-flight) mass spectrometry

1:10 and 1:20 dilutions of the tryptic digests were prepared using conditioning solution (0.1% TFA in 50% Acetonitrile). The samples were spotted in triplicates of all dilutions after mixing equal volume of the digest and matrix solution (10 mg/mL: 1 mg of Thermo Fisher Pierce α-cyano4-hydroxycinnamic acid in 100 µL of conditioning solution). The mass spectra were acquired on a Bruker Autoflex Speed MALDI-ToF mass spectrometer (Bruker Daltonics) at the proteomics facility of the Max Planck Institute of Geoanthropology. A SNAP averaging algorithm was used to obtain monoisotopic masses (C: 4.9384, N: 1.3577, O: 1.4773, S: 0.0417, H: 7.7583).

MALDIquant and MALDIquantForeign packages in R were used to convert the raw MALDI spectra in Bruker Flex format to .*mzML* file format^43,44^. The converted spectra were preprocessed in the following order using mMass software: baseline correction with a precision of 15 and a relative offset of 25; signal smoothing with the Savitzky−Golay filter using a window size of 0.3 *m/z* and two smoothing cycles. Peak picking with s/n ratio 3.5 and picking height 0.75 followed by deisotoping, generated monoisotopic peak lists. Each spectrum was then evaluated visually for the presence of 12 standard marker peaks using mMass to record their presence/absence. The observed collagen marker peptides for each artifact is recorded in Supplementary Table 2.

## Supplementary Information

Below is the link to the electronic supplementary material.

Supplementary Material 1

## Data Availability

All data generated or analyzed during this study are included in this paper and its Supplementary Information files. The mass spectra generated and analyzed for this study can be downloaded at https://doi.org/10.17632/7mhrvr5dfd.1 .
