# Supplementary material for: Characterization of raw material in textile bone instruments from the capital of the Roman Province of Lusitania (Mérida, Spain)
Source: Sci Rep. 2026 Jun 1;16:24987. doi: 10.1038/s41598-026-55935-6 (PMC13462288; doi:10.1038/s41598-026-55935-6)
Supplement: Supplementary file 1 [file 41598_2026_55935_MOESM1_ESM.pdf]

Supplementary Table 1: Summary of samples analyzed and their originating context.

Sample No.	Inventory	Type	Source	Description
MER01	8002/2326/3	Needle	Cabo Verde St., Pontezuelas St. and Octavio Augusto St.: El Disco. Chronology of the context: Roman	Funeral area
MER02	8002/00/12	Needle	Cabo Verde St., Pontezuelas St. and Octavio Augusto St.: El Disco. Chronology of the context: contemporary (residual)	Funeral area
MER03	8002/2328/5	Needle	Cabo Verde St., Pontezuelas St. and Octavio Augusto St.: El Disco. Chronology of the context: Roman	Funeral area
MER04	8002/2328/3	Needle	Cabo Verde St., Pontezuelas St. and Octavio Augusto St.: El Disco. Chronology of the context: Roman	Funeral area
MER05	8002/1102/3	Distaff	Cabo Verde St., Pontezuelas St. and Octavio Augusto St.: El Disco. Chronology of the context: Roman	Funeral area
MER06	8002/1203/9	Needle	Cabo Verde St., Pontezuelas St. and Octavio Augusto St.: El Disco. Chronology of the context: high imperial	Funeral area
MER07	8002/00/12	Needle	Cabo Verde St., Pontezuelas St. and Octavio Augusto St.: El Disco. Chronology of the context: high imperial	Funeral area
MER08	8006/163/20	Distaff	Former Campsa site: Campsa. Chronology of the context: high imperial.	Funeral area
MER09	8002/76/9	Distaff	Cabo Verde St., Pontezuelas St. and Octavio Augusto St.: El Disco. Chronology of the context: high imperial	Funeral area
MER10	8006/163/10	Spinning wheels	Former Campsa site: Campsa. Chronology of the context: High-imperial	Funeral area
MER11	8034/182/9	Needle	Pontezuelas St. / Travesía Rambla Sta. Eulalia / J.R. Mélida: Resti. Chronology of the context: imprecise	Funeral area and thermal baths
MER12	8006/163/23	Spindle	Former Campsa site: Campsa. Chronology of the context: high imperial.	Funeral area
MER13	8061/464/5	Needle	Tomás Romero de Castilla St. and Antonio Hernández Mancha St.Chronology of the context: high imperial.	-
MER14	8057/74/3	Spindle	Vía de la Plata Avenue / Albañiles St. Chronology of the context: high imperial.	Funeral area
MER15	8057/122/25	Distaff	Vía de la Plata Avenue / Albañiles St.Chronology of the context: high imperial.	Funeral area
MER16	8055/119/13	Spinning wheels	Lusitania Avenue / Barcelona St. and José Echegaray St.Chronology of the context: high imperial.	Funeral area
MER17	8067/22/232	Spatula	Cabo Verde St. and Hernán Cortés St.Chronology of the context: high imperial.	Funeral area
MER18	8067/22/33	Spatula	Cabo Verde St. and Hernán Cortés St.Chronology of the context: high imperial.	Funeral area
MER20	8101/334/12	Separator	Almendralejo, 41. Str.Chronology of the context: high imperial.	Landfill
MER21	1-00-533	Separator	Corchera RoadChronology of the context: high imperial.	Funeral area
MER22	8077/1/2	Hand loom	House of the amphitheater: Mausoleum.Chronology of the context: high imperial.	Funeral area
MER23	12005/160/1	Spinning wheels	Parejo St.Chronology of the context: high imperial.	Domestic area
MER24	12006/490/2	Distaff	"Travesía" Parejo on the corner of "Travesía" Hernán Cortés. Chronology of the context: high imperial.	Domestic area
MER25	8277/110/1	Distaff	Hernán Cortés Sr. Chronology of the context: high imperial.	Domestic area
MER26	8102/1367/459	Needle	C/ Almendralejo, 41. Mérida. Chronology of context: Vespasian-Domitian	Landfill
MER27	8102/1367/461	Distaff	C/ Almendralejo, 41. Mérida.chronology of the context: Vespasian-Domitian.	Landfill
MER28	8102/1292/283	Needle	C/ Almendralejo, 41. Mérida. Chronology of the context: mid II A.D.	Landfill
MER29	8102/1290/39	Proof of spindle wheel	C/ Almendralejo, 41. Mérida. Chronology of the context: mid II A.D.	Landfill
MER30	8102/1262/741	Distaff	C/ Almendralejo, 41. Mérida. Chronology of the context: end of III A.D.	Landfill
MER31	8102/1234/60	Needle	C/ Almendralejo, 41. Mérida. Chronology of the context: end of III A.D.	Landfill
MER32	8102/1319/1455	Distaff	C/ Almendralejo, 41. Mérida.Chronology of the context: beginning of II A.D.	Landfill
MER33	8117/220/5	Hand loom	Zone III. Antonio García Bellido St.	Landfill
MER34	8277/110/1 y 3	Distaff	Zona III. Cuartel Hernán Cortés. Fase IV.	Landfill
MER35	8277/110/4	Spindle	Zona III. Cuartel Hernán Cortés. Fase IV.	Landfill
MER36	8102/1319/1428	Needle wheel	C/ Almendralejo, 41. Mérida. Chronology of the context: begg. II A.D.	Landfill

Supplementary Table 2: Monoisotopic peaks of the sample ZooMS spectra and their assignments to the published collagen (I) peptide markers.

Plate	MALDI Plate Position	ID	Family	Regional taxon (most likely ID)	ZooMS ID (Technical ID from the spectra)	α1 508	α2 978	α2 978 (+16)	α2 244	α2 604	α1 238	α2 484	α1 688	α1 309	α2 502	α2 292	α2 889	α1 220	α2 793	α2 658	α1 910	α1 934	α1 733	α2 454	α1 586	α1 586 (+16)	α2 757	α2 757 (+16)
20240726-AC protocol	A 2,5,8	MER2	Bovidae/Cervidae	Bovidae/Cervidae	Bovidae/ Cervidae	1105.6	NA	NA				1427.7			1550.8	1648.8	1532.8		NA					NA	2883.4	NA	NA	NA
20240726-AC protocol	A 3,6,9	MER3	Bovidae	Bos taurus	Bos	1105.6	1192.6	1208.6				1427.7			1580.8	1648.8	1532		2131.1					2792.4	2853.4	2869.4	3017.4	3033.4
20240726-AC protocol	A 10,13,16	MER4	Bovidae	Bos taurus	Bos	1105.6	1192.6	1208.6				1427.7			1580.8	1648.8	1532		2131.1					2792.4	2853.4	2869.4	3017.4	3033.4
20240726-AC protocol	A 11,14,17	MER5	Bovidae	Bos taurus	Bos	1105.6	1192.6	1208.6				1427.7			1580.8	1648.8	1532		2131.1					2792.4	2853.4	2869.4	3017.4	3033.4
20240726-AC protocol	A 12,15,18	MER6	Bovidae/Cervidae	Antilopini (such as gazelle)	Antilopini	1105.6	1180.6	1196.6				1427.7			1550.8	1648.8	1532		2131.1					2792.4	2883.4	2899.4	3017.4	3033.4
20240726-AC protocol	B 1,4,7	MER7	Bovidae	Bos taurus	Bos	1105.6	1192.6	1208.6				1427.7			1580.8	1648.8	1532		2131.1					2792.4	2853.4	2869.4	3017.4	3033.4
20240726-AC protocol	B 3,6,9	MER8	Bovidae/Cervidae	Bovidae/Cervidae	Bovidae/ Cervidae	1105.6	1180.6	1196.6				1427.7			NA	NA	1532.8		NA					NA	NA	NA	NA	NA
20240726-AC protocol	B 10,13,16	MER9	Bovidae/Cervidae	Antilopini (such as gazelle)	Antilopini	1105.6	1180.6	1196.6				1427.7			1550.8	1648.8	1532		2131.1					2792.4	2883.4	2899.4	3017.4	3033.4
20240726-AC protocol	B 12,15,18	MER10	Bovidae	Bos taurus	Bos	1105.6	1192.6	1208.6				1427.7			1580.8	1648.8	1532		2131.1					2792.4	2853.4	2869.4	3017.4	3033.4
20240726-AC protocol	C 1,4,7	Mer 11	Bovidae/Cervidae	Antilopini (such as gazelle)	Antilopini	1105.6	1180.6	1196.6				1427.7			1550.8	1648.8	1532		2131.1					2792.4	2883.4	2899.4	3017.4	3033.4
20240726-AC protocol	C 2,5,8	Mer 12	Bovidae	Bos taurus	Bos/Oreotragus	1105.6	1192.6	1208.6				1427.7			1580.8	1648.8	1532		2131.1					2792.4	NA	NA	3017.4	3033.4
20240726-AC protocol	D 1,4,7	Mer 17	Bovidae	Bos taurus	Bos	1105.6	1192.6	1208.6				1427.7			1580.8	1648.8	1532		2131.1					2792.4	2853.4	2869.4	3017.4	3033.4
20240726-AC protocol	D 2,5,8	Mer 18	Bovidae	Bos taurus	Bos	1105.6	1192.6	1208.6				1427.7			1580.8	1648.8	1532		2131.1					2792.4	2853.4	2869.4	3017.4	3033.4
20240726-AC protocol	D 3,6,9	Mer 20	Bovidae	Bos taurus	Bos	1105.6	1192.6	1208.6				1427.7			1580.8	1648.8	1532		2131.1					2792.4	2853.4	2869.4	3017.4	3033.4
20240726-AC protocol	D 10,13,16	Mer 21	Bovidae	Bos taurus	Bos	1105.6	1192.6	1208.6				1427.7			1580.8	1648.8	1532		2131.1					2792.4	2853.4	2869.4	3017.4	3033.4
20240726-AC protocol	D 11,14,17	Mer 22	Bovidae	Bos taurus	Bos	1105.6	1192.6	1208.6				1427.7			1580.8	1648.8	1532		2131.1					2792.4	2853.4	2869.4	3017.4	3033.4
20240726-AC protocol	D 12,15,18	Mer 23	Bovidae	Bos taurus	Bos	1105.6	1192.6	1208.6				1427.7			1580.8	1648.8	1532		2131.1					2792.4	2853.4	2869.4	3017.4	3033.4
20240726-AC protocol	E 3,6,9	Mer 25	Elephantidae	Elephas maximus	Elephas maximus	1105.6	NA	NA				1453.7			1579.8	1568.8	1590.8		2115.1					2808.4	2853.4	2869.4	2999.4	3015.4
20240816-AC protocol Mer26-37	A 1,4,7	Mer 26	Bovidae	Bos taurus	Bos	1105.6	1192.6	1208.6				1427.7			1580.8	1648.8	1532		2131.1					2792.4	2853.4	2869.4	3017.4	3033.4
20240816-AC protocol Mer26-37	A 2,5,8	Mer 28	Bovidae	Bos taurus	Bos	1105.6	1192.6	1208.6				1427.7			1580.8	1648.8	1532		2131.1					2792.4	2853.4	2869.4	3017.4	3033.4
20240816-AC protocol Mer26-37	A 3,6,9	Mer 29	Bovidae/Cervidae	Antilopini (such as gazelle)	Antilopini	1105.6	1180.6	1196.6				1427.7			1550.8	1648.8	1532		2131.1					2792.4	2883.4	2899.4	3017.4	3033.4
20240816-AC protocol Mer26-37	A 10,13,16	Mer 30	Bovidae/Cervidae	Antilopini (such as gazelle)	Antilopini	1105.6	1180.6	1196.6				1427.7			1550.8	1648.8	1532		2131.1					2792.4	2883.4	2899.4	3017.4	3033.4
20240816-AC protocol Mer26-37	A 11,14,17	Mer 31	Aves	No published markers	No published markers																							
20240816-AC protocol Mer26-37	A 12,15,18	Mer 32	Bovidae	Bos taurus	Bos/Sylvicapra	1105.6	1192.6	1208.6				1427.7			1580.8	1648.8	1532		2131.1					2792.4	2853.4	2869.4	NA	NA
20240925-repeated samples	A 1,4,7	Mer 1	fail																									
20240925-repeated samples	A 2,5,8	Mer 13	fail																									
20240925-repeated samples	A 3,6,9	Mer 14	Bovidae	Bos taurus	Bos/ Tragelaphus/ Oreotragus	1105.6	1192.6	1208.6				1427.7			1580.8	1648.8	1532		2131.1					2792.4	2853.4	2869.4	NA	NA
20240925-repeated samples	A 10,13,16	Mer 15	fail																									
20240925-repeated samples	A 11,14,17	Mer 16	Bovidae	Bos taurus	Bos	1105.6	NA	1208.6				1427.7			1580.8	1648.8	1532		2131.1					2792.4	2853.4	2869.4	NA	NA
20240925-repeated samples	A 12,15,18	Mer 24	Bovidae	Bos taurus	Bos	1105.6	NA	1208.6				1427.7			1580.8	1648.8	1532		2131.1					NA	NA	NA	NA	NA
20240925-repeated samples	B 1,4,7	Mer 33	Bovidae	Bos taurus	Bos	1105.6	NA	1208.6				1427.7			1580.8	1648.8	1532		2131.1					NA	NA	NA	NA	NA
20240925-repeated samples	B 3,6,9	Mer 34	Bovidae	Bos taurus	Bos	1105.6	NA	1208.6				1427.7			1580.8	1648.8	1532		2131.1					NA	NA	NA	NA	NA
20240925-repeated samples	B 10,13,16	Mer 35	Bovidae	Bos taurus	Bos	1105.6	NA	1208.6				1427.7			1580.8	1648.8	1532		2131.1					NA	NA	NA	NA	NA
20240925-repeated samples	B 12,15,18	Mer 36	Bovidae/Cervidae	Bovidae/Cervidae	Bovidae/ Cervidae	1105.6	1180.6	1196.6				1427.7			1550.8	NA	NA	NA	NA					NA	NA	NA	NA	NA
20240925-repeated samples	C 1,4,7	Mer 37	Bovidae/Cervidae	Bovidae/Cervidae	Bovidae/ Cervidae	1105.6	NA	NA				1427.7			NA	NA	NA		2131.1					NA	NA	NA	NA	NA
